# Actinoporin-like Proteins Are Widely Distributed in the Phylum Porifera

**DOI:** 10.3390/md20010074

**Published:** 2022-01-15

**Authors:** Kenneth Sandoval, Grace P. McCormack

**Affiliations:** Molecular Evolution and Systematics Laboratory, Zoology, Ryan Institute & School of Natural Sciences, National University of Ireland Galway, 23 University Rd., H91 R8EC Galway, Ireland; k.sandoval1@nuigalway.ie

**Keywords:** Porifera, marine sponge, *Haliclona*, transcriptomics, actinoporins, pore-forming toxins

## Abstract

Actinoporins are proteinaceous toxins known for their ability to bind to and create pores in cellular membranes. This quality has generated interest in their potential use as new tools, such as therapeutic immunotoxins. Isolated historically from sea anemones, genes encoding for similar actinoporin-like proteins have since been found in a small number of other animal phyla. Sequencing and *de novo* assembly of Irish *Haliclona* transcriptomes indicated that sponges also possess similar genes. An exhaustive analysis of publicly available sequencing data from other sponges showed that this is a potentially widespread feature of the Porifera. While many sponge proteins possess a sequence similarity of 27.70–59.06% to actinoporins, they show consistency in predicted structure. One gene copy from *H. indistincta* has significant sequence similarity to sea anemone actinoporins and possesses conserved residues associated with the fundamental roles of sphingomyelin recognition, membrane attachment, oligomerization, and pore formation, indicating that it may be an actinoporin. Phylogenetic analyses indicate frequent gene duplication, no distinct clade for sponge-derived proteins, and a stronger signal towards actinoporins than similar proteins from other phyla. Overall, this study provides evidence that a diverse array of Porifera represents a novel source of actinoporin-like proteins which may have biotechnological and pharmaceutical applications.

## 1. Introduction

Actinoporins (APs) are proteinaceous α-pore-forming toxins originally isolated from and named after sea anemones [1]. This group of toxins typically exhibit several common characteristics, such as a common absence of cysteine residues, a high isoelectric point (>8.8), and a small size (~20 kDa) [2]. Furthermore, they comprise a compact β-sandwich flanked on each side by an α-helix, as indicated by the crystal structures of the well-studied equinatoxin II (EqT-II), stichyolysin II (Stn-II), and fragaceatoxin C (Fra-C) [3,4,5]. The molecular mechanism of cytolytic pore formation by APs has been extensively researched and appears to involve several steps, which are briefly summarized. First, lipid recognition and membrane binding are accomplished via the interfacial binding site (IBS), which features a cluster of prominent aromatic residues that bind to phosphocholine (POC) [4,6]. In particular, APs have an affinity for the POC group of sphingomyelin (SM) and are capable of discriminating between this target and other membrane lipids, such as phosphatidylcholine [6,7]. After binding to a target membrane, APs then undergo a conformational change in which the N-terminal region, containing one of the α-helices, is translocated to lie flat upon the membrane surface [8,9]. This N-terminal region is then inserted into the target membrane and undergoes further conformational change to increase the overall length of the amphipathic α-helix relative to its unbound state [10,11]. The pore is finally formed when oligomerization occurs via the recruitment of additional AP monomers, which undergo the same process in the same region of the membrane to bring about the death of targeted cells by osmotic shock [12,13]. For a more in-depth explanation on the molecular mechanisms of pore formation by APs, the reader is referred to reviews which focus on this topic [13,14,15]. The qualities of APs which allow for their membrane-binding and pore-forming activity have attracted attention regarding potential biotechnological and therapeutic applications, such as the design of immunotoxins, nanopores, adjuvants, and SM-specific probes [16,17,18,19].

Historically, sea anemones have been the primary source of APs, although similar cytolytic proteins can be found in other anthozoans [20,21]. Indeed, an exhaustive bioinformatic analysis indicated that actinoporin-like proteins (ALPs) are distributed across multiple phyla with high structural similarity despite low sequence similarity [22]. In particular, APs and ALPs have been detected in chordates (primarily teleost fish), cnidarians, molluscs, mosses, and ferns. Furthermore, a structural similarity of APs and ALPs to fungal-fruit body lectins has also been determined. A phylogenetic analysis of these identified proteins revealed four distinct groups comprising ALPs primarily found in vertebrates, hydrozoan ALPs, APs from cnidarians and plants, and fungal fruit-body lectins, all of which were proposed to comprise the actinoporin-like proteins and fungal fruit-body lectins superfamily (AF). The presence of ALP genes in non-vertebrate bilaterians has been further illuminated in studies focused on polychaetes of the genus *Glycera*, the crustacean *Xibalbanus tulumensis*, the brachiopod *Lingula anatina*, and many molluscs of the classes Gastropoda and Bivalvia [23,24,25]. Several ALPs have been functionally characterized, indicating that they can possess similar qualities to APs regarding membrane binding and cytolytic activity.

The first published example of an ALP was echotoxin II, isolated from the salivary glands of the predatory mollusc *Monoplex echo*, which also has an amphipathic N-terminal α-helix, a patch of aromatic residues, and hemolytic activity, but a specificity to gangliosides rather than SM [26]. In further contrast to APs, an ALP from the zebrafish, *Danio rerio*, possessed no cytolytic activity and its membrane-binding activity was not specific to SM [22]. Yet bryoporin, from the moss *Physcomitrella patens,* showed consistency with its close phylogenetic grouping to APs in that it also exhibited specificity for SM as well as hemolytic activity, although its biological role appears to be related to dehydration stress [27]. Similarly, clamlysin B from the bivalve *Corbicula japonica* also exhibits SM-binding and cytolytic activity [28]. Finally, the ALP HALT-1 from the cnidarian *Hydra magnipapillata*, which is phylogenetically distinct from anthozoan APs, exhibits lower hemolytic activity, the creation of larger pores, and a lower affinity to SM in comparison to EqT-II [29]. Altogether, the observation that ALPs from non-anthozoans can possess similar biochemical properties to APs supports the notion that they may also be potential targets for the aforementioned biotechnological and therapeutic applications which have been investigated for EqT-II, Stn-II, and Fra-C.

Sea sponges of the phylum Porifera are benthic, filter-feeding animals which can be found in marine and freshwater environments throughout the world. Given the niche they fill, challenges faced by these organisms include contact with pathogenic microorganisms, spatial competition with other benthic life, and predation [30,31,32]. In order to deal with these challenges, many sponges utilize complex chemical armaments which display an array of bioactivities towards targets, such as pathogens, fouling organisms and cancerous cells [33,34,35]. While a majority of these bioactivities have been attributed to small molecules, some larger proteinaceous toxins have also been identified, such as suberitine from *Suberities domuncula*, halilectin-3 from *Haliclona caerulea*, and chondrosin from *Chondrosia reniformis* [36,37,38]. In addition, sea sponges have also been shown to be a source of cytolytic pore-forming proteins, one of which is an antibacterial, perforin-like protein from *S. domuncula*, which was found to be upregulated upon exposure to lipopolysaccharide [39,40]. No ALPs have been isolated and characterized from this phylum, but a recent phylogenetic study has indicated that genes encoding for these proteins are present in the genome of the species *Oscarella pearsei* (then *O. carmela*, when its genome was sequenced) [21,41,42]. Little was reported on this ALP other than it being phylogenetically distant from both anthozoan APs, hydrozoan ALPs and mollusc ALPs. Similarly, while analyzing our transcriptome assemblies of native Irish *Haliclona* species, we noticed the presence of numerous genes encoding for proteins with the Pfam domain PF06369, representing sea anemone cytotoxic proteins. The presence of ALPs in sponges of both the classes Demospongiae and Homoscleromorpha prompted the questions of whether these proteins are widely distributed throughout the phylum and how similar they are to known APs. As discussed previously, such a quality would expand the possible biotechnological and therapeutic applications of sponges. To date, these organisms have been the subject of numerous transcriptomic and genomic studies, resulting in a wealth of public data with which to carry out such an inquiry [43]. To address these aforementioned questions and expand the knowledge of APs and ALPs, we herein present an exploration of the diversity, distribution, and predictive function of these proteins in the phylum Porifera.

## 2. Results

### 2.1. Transcriptome Sequencing and ALP Identification

After processing with fastp, approximately 472.68, 69.80, 257.97, 207.46, and 94.64 Mbp of data were acquired for *H. cinerea*, *H. indistincta*, *H. oculata*, *H. simulans*, and *H. viscosa*, respectively. Five separate transcriptomes were then assembled using the Trinity RNA-Seq assembler (Table 1). More data were available for *H. cinerea*, *H. oculata*, and *H. simulans*, which appeared to be reflected in the generally larger assembly size and higher number of true genes when compared to *H. indistincta* and *H. viscosa* (the latter two species belong to a separate species group that is phylogenetically distinct from the first three). Furthermore, this division was also apparent regarding GC content, in which the first three exhibited a value around 39%, while the latter exhibited a value of 44.5%. The total amount of translated open reading frames was largely reflected by the size of the assemblies, with *H. cinerea* yielding the most protein sequences, while *H. viscosa* yielded the least (Table 2). All five *Haliclona* transcriptomes exhibited very high completeness when assessed with the BUSCO eukaryote dataset (Table 3).

A total of 66 unique open reading frames encoding for proteins with the pfam domain PF06369 were identified in the analyzed sponge NGS resources. These ORFs were derived from 20 of the total 63 species that were screened (Table S1) Of these, 38 ORFs were determined to be complete by TransDecoder. As none of these sponge-derived proteins have been experimentally characterized, they are hence referred to as ALPs. Specifically, these complete ALPs were found in *C. orientalis* (Co), *C. varians* (Cv), *D. avara* (Da), *G. barretti* (Gb)*, H. indistincta* (Hi), *H. viscosa* (Hv), *O. pearsei* (Op), a *Scolapina* sp. (S), *S. officinalis* (So), and *T. wilhelma* (Tw). ALPs were absent from all freshwater sponge data. Their presence in an order did not necessarily translate to this being an absolute feature of said order. This is particularly exemplified by the order Haplosclerida, in which numerous paralogs were detected in the sister species *H. indistincta* and *H. viscosa*, a few in *H. amboinensis* and *H. cinerea*, and none in *H. oculata*, *H. simulans*, *H. tubifera*, or *A. queenslandica*. Only *C. varians* and *D. avara* possessed the same degree of parology as *H. indistincta* and *H. viscosa*. Of the sponge ALPs, only two from *T. wilhelma* were identified as having a signal peptide by SignalP [44]. All the complete sponge ALPs aligned most closely with cnidarian actinoporins when these were used in a blastp query against the NCBI non-redundant database (Table S2). In particular, one ALP from *H. indistincta* named Hi2 exhibited the highest sequence similarity with an actinoporin from *Haloclava producta* at 59.06% identity. It was also observed that several ALPs from incomplete ORFs most closely aligned with pore-forming proteins from other phyla, such as coluporins and tereporins from the Mollusca. No ALPs were detected in the screened genomes or transcriptomes of choanoflagellates, ctenophores, or placozoans. The theoretical isoelectric point of complete sponge ALPs ranged from 4.66 to 9.46, whereas the average molecular weight ranged from 14,153.27 to 33,419.71 Da (Table S3).

### 2.2. Structural Prediction

**Figure 1:**
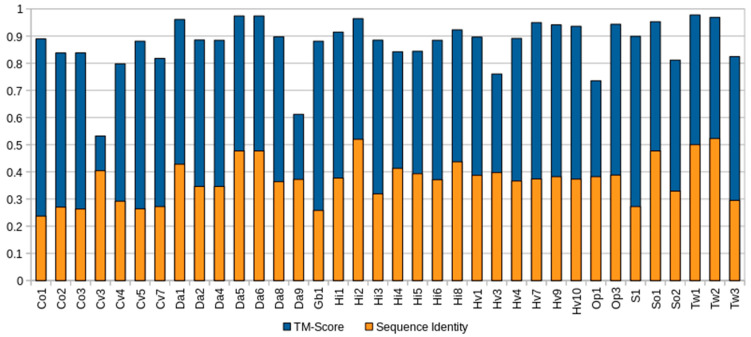
Comparison of complete sponge ALPs with EqT-II. Blue represents the TM-score of all predicted sponge ALP structures via Phyre2 with the crystal structure of EqT-II (1IAZ). Orange represents the sequence identity of the aligned region of the two structures. The orange bar representing percent identity is overlaid upon the blue bar representing TM-score. Abbreviations are as follows: Co, *C. orientalis*; Cv, *C. varians*; Da, *Dysidea avara*; Hi, *H. indistincta*; Hv, *H. viscosa*; Op, *O. pearsei*; *S, Scopalina* sp.; So, *S. officinalis*; Tw, *Tethya wilhelma*. The protein Sc1 from *S. carteri* is excluded due to having unknown residues. Sequence identity is expressed on a scale of 0–1 rather than as a percentage.

In contrast to the low-to-modest sequence similarity many sponge ALPs showed in relation to actiniarian APs (27.70–59.06%) (Table S2), homology modeling with Phyre2 indicated that the aligned predicted structure of all complete sponge ALPs was highly similar to that of Stn-II and EqT-II, with confidence values ranging from 97.3% to 100%. Quantification of the similarity between the predicted sponge ALP models and the crystal structure of EqT-II via TM-align showed that all produced models had a TM-align value above 0.5, indicating that the structural similarity was not random (Figure 1), whereas the RMSD values ranged from 0.54 to 1.74 (Å) (Table S4).

**Figure 2:**
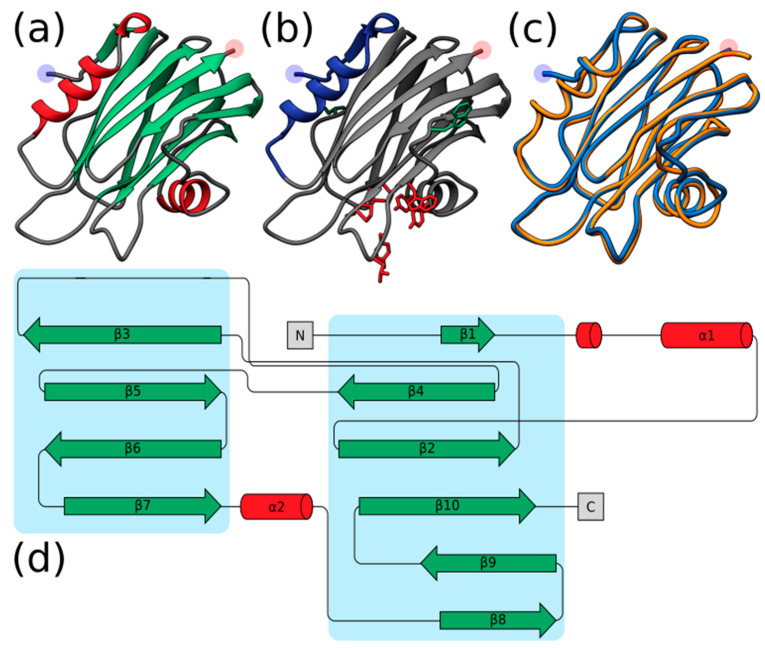
(**a**) Predicted structure of Hi2 by Phyre2. Red represents α-helices. Green represents β-sheets. (**b**) Significant functional residues of Hi2. Blue represents the N-terminal α-helix associated with pore formation. Red represents the residues of the interfacial binding site. Green represents the residues associated with oligomerization. (**c**) Structural alignment of Hi2 in blue upon EqT-II chain A (1IAZ) in orange. For all predicted structures blue and red highlights represent the N- and C-terminus, respectively. (**d**) Protein topology plot of Hi2 with the same color scheme as (**a**). The size of the topology plot was manually reduced to be more compact.

It must be noted, however, that several sea sponge ALPs exhibited structural inconsistencies with the expected AP skeleton, such as the lack of an N-terminal α-helix. Hi2 exhibited one of the highest TM-scores at 0.96406. The quality of the predicted model for Hi2 was further supported with a ProQ3D S-score of 0.697 (0.5–1.0 representing a good model; Arne Elofsson, personal communication) and a ModFOLD8 *p*-value of 3.772 × 10^−6^ (less than a 1/1000 chance that the model is incorrect). As can be seen by the structure generated by Phyre2, Hi2 shares many characteristics typical of cnidarian actinoporins, such as comprising a β-sandwich flanked by two α-helices (Figure 2a,d). Furthermore, the localization of the interfacial binding site can also be observed (Figure 2b). In general, the predicted structure of Hi2 appears to overlay well with the crystal structure of EqT-II chain A (1IAZ) (Figure 2c).

### 2.3. Multiple Sequence Alignment and Residue Analysis

**Figure 3:**
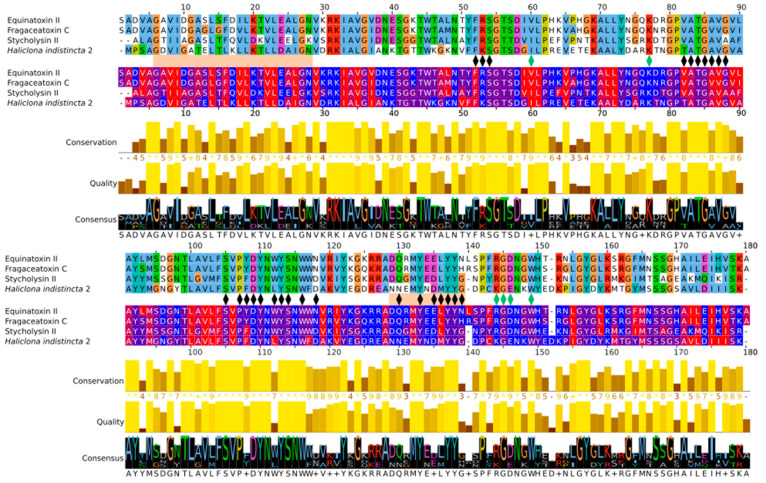
Multiple sequence alignment of Hi2 with EqT-II, Fra-C, and Stn-II. The top alignment represents Clustalx color coding. The bottom alignment represents hydrophobicity color coding. Between these two alignments, black markers represent residues important for membrane binding, green markers represent residues important for oligomerization, and orange rectangles represent the α-helices of EqT-II (the final length of the N-terminal α-helix after a conformational change and insertion into the target membrane is presented) [10,12,13,15,45,46].

Due to its high sequence similarity with cnidarian actinoporins, Hi2 was chosen for further in-depth analyses to determine whether it exhibited the same membrane-binding and pore-forming activities. A multiple sequence alignment of Hi2 with the final mature peptides of the well-studied equinatoxin II (EqT-II; P61914), fragaceatoxin C (Fra-C; B9W5G6), and stichyolysin II (Stn-II; P07845) indicated a percent identity of 50.56%, 50.00%, and 51.41%, respectively (Figure 3). Despite these modest values, the alignment illustrated a high degree of conservation regarding residues and motifs critical for the functional activities of actinoporins [15]. For example, a majority of the residues associated with the interfacial binding site in Hi2 are consistent with those of EqT-II, Fra-C, and Stn-II [6]. In addition, Hi2 possesses the conserved residue Tyr112, which is critical for SM recognition; however, a substitution of Leu for Trp at residue 111 is also observed. Furthermore, the presence of Ser53, Val86, Ser104, Pro106, Trp115, Tyre132, Tyr136, and Tyr137 are consistent with the POC binding site found in cnidarian APs [4,6,15], and the conserved P-[WYF]-D binding motif found in this region of APs is also present in Hi2 at residues 106–108 [22]. Oligomerization of actinoporin monomers upon the cell membrane is another crucial step towards pore formation and is known to be influenced by an Arg–Gly–Asp motif. Hi2 shows inconsistency with this motif, as it instead possesses Lys142, Gly143, and Glu144. However, Hi2 possesses the residue Lys76, which is consistent with similar residues associated with oligomerization in other APs [45]. The presence of Ile59 and Trp147 are also partially consistent with residues of Fra-C, associated with oligomerization and protein–protein interaction between protomers; the observed substitution of Ile for Val at this site can be seen in Stn-II [13,46]. Unlike most cnidarian APs, Hi2 exhibits the presence of cysteine at residue 141, but such a characteristic is not unheard of in these proteins [21].

**Figure 4:**
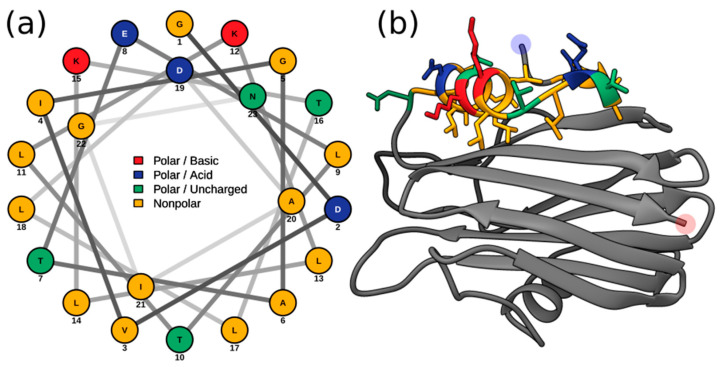
(**a**) Edmundson peptide helical wheel projection of residues 5–28 from Hi2. (**b**) Residues 5–28 of the Phyre2 predicted structure of Hi2 colored in the same manner as (**a**). Blue and red highlights represent the N- and C-terminus, respectively.

Membrane penetration and pore formation by oligomerized APs is achieved by their respective amphipathic N-terminal α-helices. In EqT-II, the N-terminal region undergoes a conformational change, producing an α-helix comprising residues 6–28 which is capable of spanning a target membrane [10]. These residues correspond to a conserved N-terminal glycine and C-terminal asparagine of the α-helix, which are also present in Fra-C and Hi2. Within this region, Hi2 also showed consistency with several previously determined highly conserved hydrophobic residues (Val7, Ile8, Leu13, Leu18, Leu22, and Ile25), as well as Arg30, which is associated with the insertion of the α-helix into the target membrane [20]. Using these sequence boundaries allowed for the construction of an Edmundson peptide helical wheel of the predicted Hi2 N-terminal α-helix after a hypothetical conformational change (Figure 4a). Consistent with the amphipathic nature of the N-terminal α-helix of EqT-II, Fra-C, and Stn-II, a side comprising a majority of polar amino acids opposite another comprising a majority of nonpolar amino acids can be seen in that of Hi2. Furthermore, the hydrophobic moment of Hi2, a measure of helix amphipathicity, was calculated to be 0.384 µH, which is comparable to that of EqT-II at 0.337 µH. The N-terminal α-helix of Hi2 exhibits hydrophobicity of 0.607 and a net charge of 0. The two faces of the N-terminal α-helix prior to a hypothetical conformational change also display a hydrophobic and hydrophilic side, which are, respectively, oriented towards and away from the rest of the protein (Figure 4b).

### 2.4. Phylogenetic Analysis

**Figure 5:**
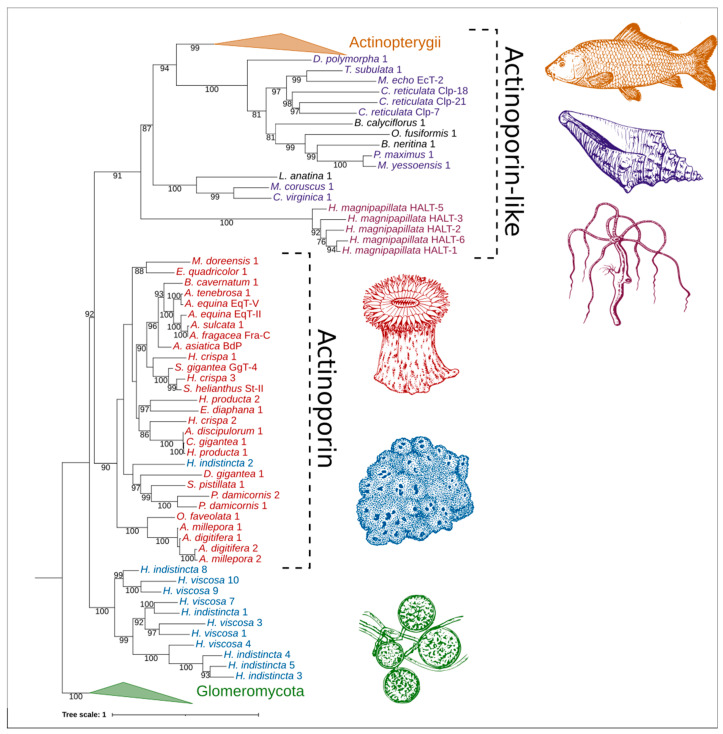
Phylogenetic tree of ALPs from the genus *Haliclona* and similar proteins. Numbers on nodes represent maximum likelihood bootstrap values above 75%. The color scheme is as follows: green represents ALPs from fungi of the class Glomeromycota; blue represents ALPs from *Haliclona* sp.; red represents APs from cnidarians of the class Anthozoa; reddish-purple represents ALPs from cnidarians of the class Hydrozoa; purple represents ALPs from the phylum Mollusca; orange represents ALPs from fish of the class Actinopterygii; black represents ALPs from miscellaneous phyla. Abbreviations are as follows: EqT, equinatoxin; Fra, fragaceatoxin; St, sticholysin; BdP, bandaporin; GgT, gigantoxin; HALT, hydra actinoporin-like toxin; EcT, echotoxin; Clp, coluporin. The glomeromycete vector image is a reproduced copy of the high-resolution image 4341-2_P1 of *Rhizophagus irregularis* (Błaszk., Wubet, Renker, and Buscot) C. Walker and A. Schüßler derived from AAFC/CCAMF (https://agriculture.canada.ca/en/agricultural-science-and-innovation/agriculture-and-agri-food-research-centres-and-collections/glomeromycota-vitro-collection-ginco/catalogue-arbuscular-mycorrhizal-fungi-strains-available-glomeromycetes-vitro-collection) (accessed on 25 November 2021). The sea sponge, sea anemone, seashell, and carp vector images were derived from those of Pearson Scott Foresman under a CC0 1.0 license (https://commons.wikimedia.org/wiki/File:Sponge_(PSF).png; https://commons.wikimedia.org/wiki/File:Anemone_2_(PSF).png; https://commons.wikimedia.org/wiki/File:Seashell_3_(PSF).png; https://commons.wikimedia.org/wiki/File:Carp_(PSF).jpg) (accessed on 26 November 2021). The *Hydra vulgaris* vector image was derived from the Freshwater and Marine Image Bank at the University of Washington under a CC0 1.0 license (https://commons.wikimedia.org/wiki/File:FMIB_50097_Hydra_vulgaris.jpeg) (accessed on 26 November 2021).

The relatedness between AP/ALP sequences from the two *Haliclona* species in which they were present, AP sequences from cnidarians, and ALP sequences from other taxa was visualized with an initial maximum likelihood tree (Figure 5). Here we find that the sequence Hi2 from *H. indistincta* nestles well within the AP clade from cnidarians, but no sequence of this type was found in its close sister species, *H. viscosa*. Additional (ALP) sequences from *H. indistincta* and its sister species *H. viscosa* form a distinct and highly supported clade well outside that of the cnidarians, indicating radiation from an additional AP/ALP copy in the ancestor of that species group. This clade is distinct from the other animal ALPs, which also form a monophyletic grouping supported by 91 BP.

**Figure 6:**
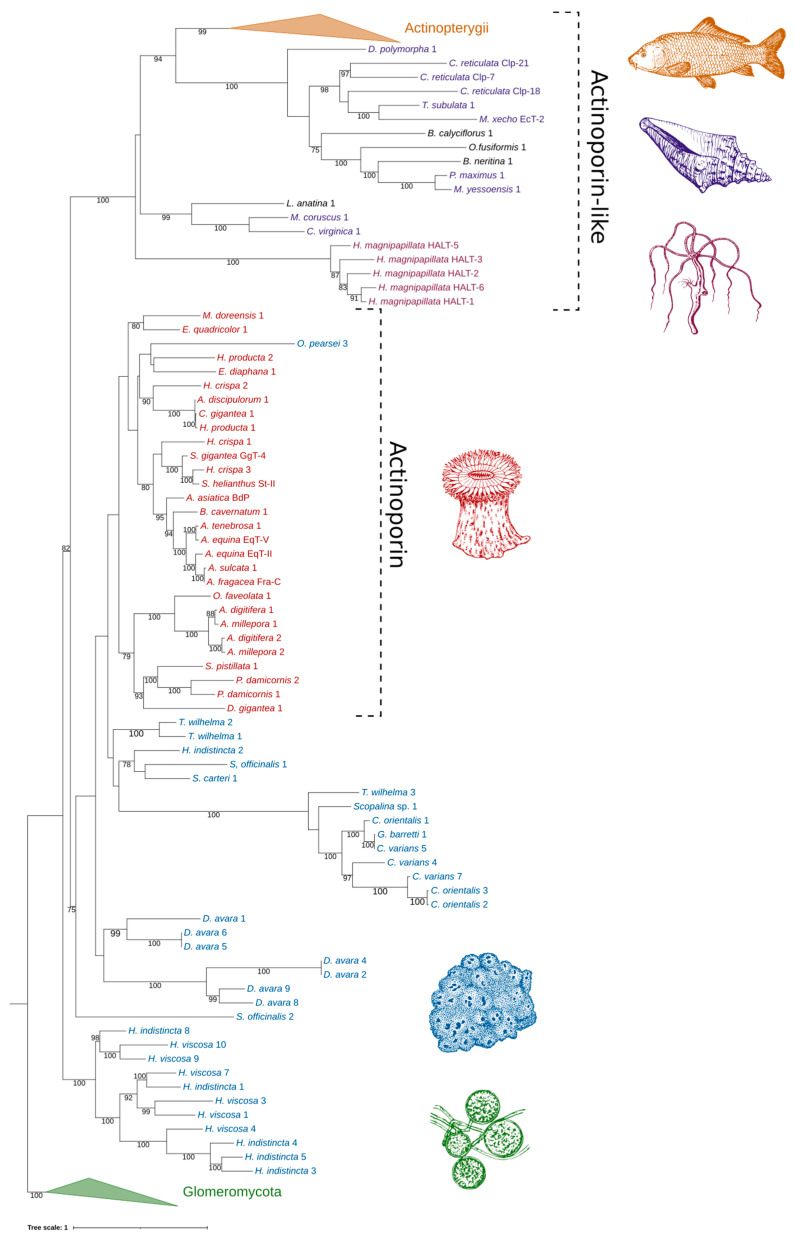
Phylogenetic tree of ALPs from sea sponges and similar proteins. Numbers on nodes represent maximum likelihood bootstrap values above 75%. Blue represents sea sponge AP/ALPs. All other color schemes as well as image acknowledgements are identical to those of Figure 5.

When all available AP/ALP sequences from Porifera are added to the analyses, the high number of paralogs present in the dataset obscure the phylogenetic signal and reduce confidence in generating an accurate phylogeny. However, the reconstructed maximum likelihood tree provides insight into the sequence similarity and potential relatedness of the sea sponge ALPs, as well as similar proteins from other phyla. Four distinct groups with strong bootstrap support were produced: (1) ALPs from fungi of the class Glomeromycota (used as outgroup), (2) the majority of sequences from the genus *Haliclona*, (3) anthozoan APs with other sponge ALPs, and (4) ALPs from other invertebrates and chordates (Figure 6). The strong grouping of the *Haliclona* ALPs, independent of all other sequences and sitting at the base of the tree, was a consistently observed phenomenon while testing other alignments for the reconstruction of a final tree (data not shown). These are a sister group to all other APs and ALPs in the dataset. The remaining APs and ALPs form a monophyletic grouping within which there are two clades; one, supported by 100 BP, consisting of the freshwater Hydra and the bilaterians, the other containing the marine cnidarians and sponges (75 BP). The presence of a large number of other sponge sequences has the effect of pulling the Hi2 sequence outside the cnidarian clade, which itself is no longer supported by bootstrapping. Relationships between the other sponge sequences are unclear, as indicated by the low bootstrap support of internal nodes. This is particularly exemplified by the likely spurious placement of Op3 within the clade of anthozoan APs. Frequently, it was observed that additions or subtractions of sequences in the alignment would result in this protein—along with Hi2, Sc1, So1, Tw1, and Tw2—being shifted in and out of the cnidarian AP group. The main exception to this is a second strongly supported clade consisting of ALPs from the genera *Cliona*, *Geodia*, *Scopalina*, and *Tethya*. While this clade may move relative to other sponge sequences, the clade remained intact and distinct from the anthozoan APs. Two groups of *D. avara* sequences were present, both highly supported, but not always remaining together on trees, depending on the comparative sequences included. They were also always distinct from anthozoan APs. Despite having a high sequence similarity to both anthozoan APs and sponge ALPs, those derived from plants, such as bryoporin, were only found to introduce additional noise into the data without significantly changing tree topology and were thus excluded, being hypothesized to be the result of a horizontal gene transfer event (data not shown) [27].

## 3. Discussion

Cytolytic pore-forming toxins are widely distributed throughout prokaryotic and eukaryotic life and often function as immunological defenses in the latter [47,48]. The proposed AF superfamily includes α-pore-forming toxins derived from diverse eukaryotic lineages with similar predicted protein structure despite low sequence similarity [22]. Members of this family include anthozoan APs, plant APs, hydrozoan ALPs, ALPs from other animals, and fungal fruit-body lectins. Herein, new additions from the phylum Porifera are proposed to belong to the AF superfamily, as members of the classes Demospongiae, Homoscleromorpha, and Calcarea have been shown to possess genes encoding for ALPs. Many functionally characterized AF proteins appear to serve a role in envenomation as they have been localized in the nematocysts of cnidarians and the salivary glands of predatory molluscs [25,26,29,49]. However, AF proteins appear to also have functions other than envenomation, as indicated by their presence in the mesenteric filaments of cnidarians, as well as organisms which do not perform this process, such as bivalves [28,49]. With this in consideration, it is not entirely unusual to observe the presence of these proteins in non-venomous animals, such as sponges, in which they may serve a different ecological function via a similar molecular mechanism.

When considering the taxonomic distribution of ALPs in sponges, no clear pattern can be discerned. The majority of the identified ALPs were derived from the Demospongiae and more specifically from the orders Axinellida, Bubarida, Clionaida, Dendroceratida, Dictyoceratida, Haplosclerida, Poecilosclerida, Suberitida, Tethyida, and Tetractinellida, but not Chondrillida, Spongillida, or Verongiida. However, further analysis at the genus level indicated that these proteins appear to have been lost in species closely related to those which possess ALPs. This is particularly exemplified by the genera *Haliclona* and *Geodia*, in which the transcriptomes of numerous species have been reported [43,50]. Furthermore, ALPs appear to be distributed throughout the phylum Porifera, as several were identified in the classes Homoscleromorpha and Calcarea, but not in Hexactinellida. However, two caveats should be considered regarding these observations. The first is that the high prevalence of these proteins in the Demospongiae is most likely due to sampling bias, as transcriptomes of this class have been disproportionately sequenced compared to the other three. Second, as most of the data analyzed in this study are derived from transcriptomes, a lack of gene expression or sequencing depth cannot be ruled out as an explanation for these genes not having been identified in a species. That said, the absence of ALPs in the *Amphimedon queenslandica* genome does appear to indicate that the loss of ALP genes has occurred in the order Haplosclerida, which may be an explanation for their absence in other species [51].

The predicted structure of most identified ALPs from sponges exhibited a high degree of aligned structural similarity to anthozoan APs, despite a low to moderate sequence similarity. Such an observation is common in studies of AF proteins from other organisms [22,25]. That said, the observed high aligned structural similarity does not necessarily equate to these sea sponge ALPs having the same membrane-binding and pore-forming capabilities. This is particularly exemplified by the observation that the N-terminal region of sea sponge ALPs appears to vary greatly. For example, this region is fully present in Hi2, truncated in Hi3, and completely absent in Hi4. Such a quality is not unique to sea sponge ALPs, as can be seen by analyzing those of hydrozoans [29]. Similarly, an incomplete N-terminal α-helix was also observed on the ALP Dr1 from *D. rerio* and was hypothesized to influence the lack of pore-forming capabilities and specificity towards SM exhibited by the protein [22]. With this in mind, many of the identified ALPs from sea sponges may serve functions other than those associated with well-characterized APs, such as EqT-II. In contrast, the higher similarity of Hi2 to anthozoan APs at both a sequence and structural level could indicate that it is capable of SM recognition and pore formation, which prompted a further analysis on this concept.

A multiple sequence alignment of Hi2 with the model APs EqT-II, Fra-C, and Stn-II indicated that this predicted protein shares numerous conserved residues associated with the fundamental processes of lipid recognition and membrane binding, insertion of the N-terminal α-helix into the target membrane, and oligomerization allowing for pore formation. The presence of a patch of aromatic residues (Tyr114, Trp117, Tyr134, Tyr138, and Tyr139) in Hi2 is consistent with the IBS observed in the model APs EqT-II, Fra-C, and Stn-II. The major observed deviation is that a substitution of leucine for tryptophan occurs at residue 113 of Hi2. The importance of the equivalent residue in EqT-II, Trp112, in membrane binding and SM recognition has been exemplified by studies in which this residue was mutated to phenylalanine or subject to ^19^F NMR studies [52,53]. This substitution also appears to be prevalent in nature, as it exists in numerous anemone APs, as well as the ALP Dr1 from *D. rerio* [20,22]. Furthermore, a mutant of EqT-II containing this substitution exhibited similar SM specificity to the wild-type protein [6]. For these reasons, this substitution in Hi2 is not expected to significantly inhibit any possible membrane-binding and SM-recognition capabilities of the protein. While at a lower level of conservation the N-terminal region of Hi2 also appears to form an α-helix of similar length to EqT-II, Fra-C, and Stn-II. The notion that this α-helix is capable of inserting itself into a target membrane is supported by the amphipathic nature of the predicted helical wheel and its hydrophobic moment comparable to previously analyzed AP N-terminal α-helices [20]. Finally, residues associated with oligomerization were somewhat consistent between Hi2 and the model actinoporins. While Hi2 showed conservation at residues Lys76, Ile59, and Trp147, whose equivalents in anthozoan APs are associated with oligomerization, its RGD motif was another site of substitution [12,13,45,46]. Hi2 instead shows a substitution of Lys for Arg and Glu for Asp. That said, Lys and Glu are of a similar charge and hydrophobicity to Arg and Asp, respectively, and may allow for the retention of the oligomerization function [12]. Furthermore, this Lys substitution has been observed in natural actinoporins [20]. Hi2 and many other sponge ALPs also exhibited an acidic pI (Supplementary Table), which is uncharacteristic of the typically basic anthozoan APs [15,54]. This observation is not entirely unusual, however, as there is a precedent for acidic APs derived from anthozoans [20,54,55].

The identification of ALPs from glomeromycete fungi with high sequence similarity to known APs supports the previous notion of a pre-metazoan origin of these proteins [22]. The presence of these genes in fungi, sponges, and cnidarians, and their absence in choanoflagellates, ctenophores, and placozoans, could possibly be explained by a series of gene losses occurring throughout their history. However, additional assemblies from these organisms should be assessed prior to making this conclusion. Furthermore, the observation that many species of sea sponge were the source of numerous ALP isoforms may be an indication that duplication of this gene is a common event in this phylum, similar to the situation in cnidarians and molluscs [25,54]. This observed diversification paired with the fact that many sponge ALPs were derived from transcriptomes indicates that these are not simply genomic relics but do play some sort of functional role in sponges. In the phylogenetic analysis, the sea sponge ALPs were not found to cluster together in a clear monophyletic group, which appears to further exemplify the notion of a high degree of divergence of these proteins in the porifera. However, based on the strong signal that numerous sponge ALPs share with anthozoan APs, to the point of being grouped together, and considering the higher sequence similarity many sponge ALPs have with cnidarian APs, several of these proteins from sponges may instead be classifiable as APs.

## 4. Materials and Methods

### 4.1. Sample Collection

**Figure A1:**
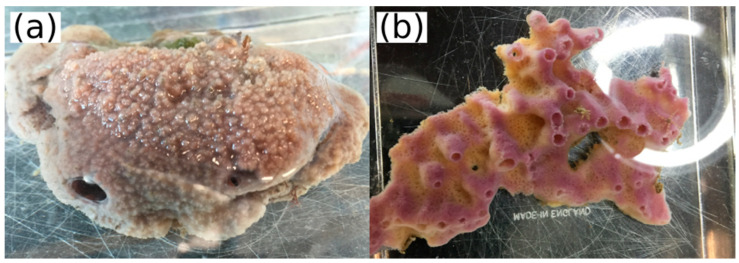
(**a**) *Haliclona indistincta* (MIIG1388). (**b**) *Haliclona viscosa* (MIIG1389).

*Haliclona indistincta* (MIIG1388; Appendix A Figure A1a) was collected at Corranroo on 17/5/2019 and *H. viscosa* (MIIG1389 and MIIG1390; Figure A1b) was collected at Bridges of Ross on 1/8/2019. The following sample processing protocol was applied to both species. Visible epibionts were removed. The sponges were rinsed in sterile artificial seawater. The sponges were then dissected into ~1 cm^3^ pieces and flash-frozen with liquid nitrogen. Samples were stored at −70 °C until further use. Voucher specimens were stored in ethanol.

### 4.2. RNA Extraction

A ~1 cm3 piece of flash-frozen sponge tissue was submerged in 500 µL of Trizol in a 2 mL microcentrifuge tube. The tissue was semi-homogenized by hand with a plastic pestle. An additional 500 µL of Trizol was then added to the sample. The sample was mixed by gently inverting five times and allowed to incubate at room temperature for 5 min. The sample was then inverted and vortexed with a VWR Analogue mini vortex mixer at maximum speed for 2 min. A volume of 100 µL BCP was added to the sample. The sample was mixed by hand for 20 s and then vortexed for 10 s with a VWR Analogue mini vortex mixer at maximum speed. The sample was incubated at room temperature for 5–10 min. The sample was then centrifuged at 16,000 g for 15 min at 6 °C. The clear, aqueous layer at the top was transferred to a fresh microcentrifuge tube. RNA was purified by first adding 500 µL of 100% isopropanol to the aqueous phase sample. The sample was then inverted and vortexed with a VWR Analogue mini vortex mixer at maximum speed for 2 min. The sample was left to incubate for 10 min at room temperature. The sample was then centrifuged at 16,000 g for 15 min at 4 °C. The supernatant was discarded and 1 mL of 75% EtOH was added to the RNA pellet. The pellet was disrupted by vortexing. The RNA sample was then centrifuged for 5 min at 4 °C at 7500 g. The EtOH was carefully removed without disturbing the pellet. The washing with 75% EtOH was repeated once. The RNA sample was allowed to air dry until the edges of the pellet were visible. Finally, the pellet was resuspended in 100 µL molecular-grade water. The RNA sample was kept frozen at −70 °C until further use. A subsample of each RNA extraction was used for quality and quantity assessment on a 2100 Bioanalyzer RNA Eukaryotic Chip.

### 4.3. Transcriptome Sequencing

Samples were sent to Macrogen, Inc. for the preparation of Illumina TruSeq Stranded mRNA libraries from poly-A selection, with insert sizes of 150 bp. The libraries were sequenced on a Novaseq, with a targeted 40 million reads per sample.

### 4.4. Transcriptome Assembly

Previously sequenced raw cDNA Illumina reads of *H. cinerea* (culture held at Carna Marine Research Station), *H. oculata* (MIIG1250 and MIIG1251), *H. indistincta* (MIIG1093, MIIG1094, MIIG1095), and *H. simulans* (MIIG1248 and MIIG1249) were acquired from Prof. Grace P. McCormack and Dr. Jose Maria Aguilar-Camacho for use in this study (personal communication) [43].

Raw cDNA reads of *H. cinerea*, *H. indistincta*, *H. oculata*, *H. simulans*, and *H. viscosa* were processed with fastp version 0.2 using default settings to remove adapters and low-quality regions [56]. The processed reads were then assembled with Trinity version 2.8.5 using default settings [57]. Reads were pooled so that one transcriptome per species was assembled. Isoforms and low-expressed transcripts were retained in the final assembly. The longest translated open reading frames per transcript were extracted using TransDecoder version 5.5.0 [58]. Homology searches using these open reading frames as a query against the SwissProt database (accessed on 14 January 2020) with BLASTp version 2.9.0 [59,60], as well as the Pfam database (accessed on 14 January 2020) with HMMER version 3.2.1 [61,62], were performed. Significant homologous alignments were used to guide TransDecoder in identifying additional open reading frames. The completeness of the transcriptomes was then assessed using BUSCO version 5.1.2; specifically, the assemblies were queried against the latest version of the eukaryota_odb10 dataset (downloaded 13/4/2021) [63]. Transcriptome assembly, open reading frame extraction, BUSCO analysis, and the generation of the maximum likelihood trees were performed with an account at the Leibniz Supercomputing Centre.

### 4.5. Identification of Novel Actinoporin-like Proteins from Sea Sponges

While analyzing the output of the homology search against the Pfam database, it was noticed that numerous translated protein sequences possessed the Pfam domain PF06369 representing sea anemone cytotoxic proteins, such as actinoporins. Due to the biotechnological potential of actinoporins, this prompted the screening of all publicly available sea sponge genomes [41,51,64,65,66,67,68] and transcriptomes [42,50,65,68,69,70,71,72,73,74,75,76,77,78,79,80,81,82,83,84,85,86] to see if other members of the phylum also encoded ALPs in their genome. If transcriptome assemblies were not provided in the original publication, the data were assembled in the same manner as the Irish *Haliclona*. The *Haliclona* ALPs were used as queries in a tblasn search against the other sponge assemblies with an e-value cutoff of 1e-4. The longest open reading frames were then extracted from the hits using TransDecoder version 5.5.0 [58]. These protein sequences were screened against the Pfam database (accessed on 14 January 2020) with HMMER version 3.2.1 [61,62] and only those indicated as possessing domain PF06369 were retained for further analysis. These identified sponge ALPs were also screened against the NCBI Conserved Domain Database to confirm that PF06369 was the primary conserved domain [87]. To explore the possible evolutionary origin of ALPs in animals, the genomes of the choanoflagellates *Monosiga brevicollis* and *Salpingoeca rosetta*, the ctenophores *Mnemiopsis leidyi* and *Pleurobrachia bachei*, and the placozoans *Trichoplax adhaerens* and *Hoilungia hongkongensis* were also screened using EqT-II as a query [88,89,90,91,92,93]. Furthermore, nineteen choanoflagellate transcriptomes were also screened in a similar manner [94].

### 4.6. Sequence Analysis and Structural Prediction

All identified sea sponge ALPs were used as a query against the NCBI non-redundant protein database to identify the closest homologous sequence [95]. SignalP 5.0 was used to identify the presence of signal peptides in sea sponge ALPs contained within a complete ORF [44]. The isoelectric point and molecular weight of complete, mature sponge ALPs were determined using the compute pI/Mw tool of ExPASy [96]. Protein structure prediction of all sponge ALPs was performed using Phyre2 Suite version 5.1 [97]. The quality of the predicted protein structure for Hi2 was assessed with ProQ3D and ModFOLD8 [98,99]. Structural alignment of sponge ALPs upon the crystal structure of chain A from EqT-II (1iaz) was performed with TM-Align [100]. Protein structures were visualized using UCSF Chimera version 1.15 [101]. A protein topology plot was created using Pro-origami [102]. A multiple sequence alignment of Hi2, EqT-II (P61914), Fra-C (B9W5G6), and Stn-II (P07845) was performed with MAFFT v7.490, with the L-INS-i alignment method using default settings [103]. The multiple sequence alignment was then visualized using Jalview version 2.11.1.4 [104]. Analysis of the N-terminal α-helix of Hi2 and generation of an Edmundson wheel projection were accomplished using HeliQuest and NetWheels [105,106]. RNA 3D structure prediction was performed with 3dRNA v2.0 [107].

### 4.7. Phylogenetic Analysis of Actinoporin-like Proteins from Sea Sponges

Sea sponge ALPs derived from complete ORFs were chosen for multiple sequence alignment and phylogenetic analysis. All alignments were performed using MAFFT 7.490, with the L-INS-i alignment method using default settings [103]. APs were represented by well-characterized actiniarian proteins, such as the aforementioned EqT-II, Fra-C, and Stn-II, as well as those from stony and soft corals. ALPs were represented by the series of HALT proteins from H. magnipapillata. It was observed that in general the sea sponge ALPs most consistently aligned with sequences from sea anemones, fungi of the class Glomeromycota, and teleost fish, in that order. To get more sequences for the phylogenetic tree, all complete sponge ALPs were queried against the NCBI nr database against these three taxonomic groups, as well as against molluscs and invertebrates which did not fall under these aforementioned phyla. The top hit from each category for each sponge sequence was then retrieved. This resulted in seven groups of sequences: glomeromycete fungi, sponges, anthozoan cnidarians, hydrozoan cnidarians, molluscs, miscellaneous invertebrates, and teleost fish (Table S5). All signal peptides were removed prior to alignments using SignalP version 5.0 [44]. Each of these groups were separately aligned to the mature sequences of EqT-II, Fra-C, and Stn-II. The individual alignments were then trimmed corresponding to the boundaries of EqT-II, Fra-C, and Stn-II using using Jalview version 2.11.1.4 [104]. Several sponge ALPs, while complete, where excluded due to being excessively truncated compared to EqT-II or introducing significant gaps. All trimmed sequences were then pooled together and once more aligned. Maximum likelihood trees were constructed in IQ-TREE version 2.1.4 with 1000 bootstrap pseudoreplicates with the intention of visualizing the degree of similarity the sponge ALPs had to APs and ALPs from other phyla [108]. The resulting phylogenetic trees were modified using the Interactive Tree of Life (iTOL) v6 [109].

### 4.8. Generation of Figures

Several figures were further modified using the GNU Image Manipulation Program 2.10.28 [110] and Inkscape 0.92 [111].

## 5. Conclusions

Sea sponges, like some other invertebrates, are a source of ALPs. These proteins exhibit a high degree of predicted structural similarity as well as a phylogenetic signal to APs from cnidarians. One AP, Hi2, also possesses a majority of conserved residues associated with essential functions, including the recognition of SM, binding to membranes, oligomerization, and the formation of pores. While their ecological role in sponges remains to be determined, the aforementioned qualities encourage the exploration of these proteins for the biotechnological applications which have been proposed for anthozoan APs.

## Figures and Tables

**Table 1:** Trinity assembly statistics for the five *Haliclona* transcriptomes.

Species	Total (Mbp)	Number of Contigs	Number of Trinity ‘Genes’ Excluding Isoforms	Contig N50 (Kbp)	GC (%)
*H. cinerea*	156.12	123,111	64,261	2.81	39.59
*H. indistincta*	106.87	101,413	48,788	2.03	44.10
*H. oculata*	142.18	122,855	70,008	2.46	38.56
*H. simulans*	104.12	106,366	55,501	1.89	39.87
*H. viscosa*	94.09	105,831	59,949	1.73	44.50

**Table 2:** TransDecoder open reading frame statistics for the five *Haliclona* transcriptomes.

Species	Total (aa)	Number of Complete ORFs	Number of 5′ Partial ORFs	Number of 3′ Partial ORFs
*H. cinerea*	34,243,765	58,606	14,476	6129
*H. indistincta*	26,553,534	29,232	15,794	7290
*H. oculata*	30,530,592	49,528	16,191	5687
*H. simulans*	24,643,377	31,081	16,112	7561
*H. viscosa*	22,299,218	24,435	12,776	7166

**Table 3:** BUSCO eukaryotic score for the five *Haliclona* transcriptomes.

Species	Complete (%)	Single (%)	Duplicate (%)	Fragmented (%)	Missing (%)
*H. cinerea*	97.6	23.9	73.7	0.8	1.6
*H. indistincta*	99.6	45.1	54.5	0.0	0.4
*H. oculata*	98.5	31.4	67.1	0.4	1.1
*H. simulans*	97.6	38.0	59.6	2.0	0.4
*H. viscosa*	95.3	55.3	40.0	4.3	0.4

## Data Availability

The raw RNA-seq reads are available at the NCBI BioProject PRJNA795170. Transcriptome assemblies, sea sponge ALP sequences, predicted protein structures, multiple sequence alignments, and maximum likelihood trees are available at Mendeley (https://data.mendeley.com/datasets/w9t6zsjjb7/1, accessed on 14 December 2021).

## Supplementary Materials

The following are available online at www.mdpi.com/article/10.3390/md20010074/s1, Table S1. Presence; Table S2. Blastp nr; Table S3. pI-Mw; Table S4. TM-align; Table S5. Trees.
